# PCR-NGS技术检测异基因造血干细胞移植前FLT3-ITD基因突变负荷的临床应用价值

**DOI:** 10.3760/cma.j.cn121090-20250325-00147

**Published:** 2025-12

**Authors:** 雅聪 邵, 赟翔 张, 伟玲 孙, 皓 陆, 传和 江, 璐祥 王, 佳瑜 黄, 子璐 张, 海洋 陆, 增凯 潘, 文伟 朱, 晓霞 胡

**Affiliations:** 1 上海交通大学医学院附属瑞金医院血液科，上海血液学研究所，国家转化医学中心，上海 200025 National Research Center for Translational Medicine, State Key Laboratory of Medical Genomics, Shanghai Institute of Hematology, Ruijin Hospital, Shanghai JiaoTong University School of Medicine, Shanghai 200025, China; 2 上海中医药大学附属岳阳中西医结合医院血液科，上海 200437 Department of Hematology, Yueyang Hospital of Integrated Traditional Chinese and Western Medicine, Shanghai University of Traditional Chinese Medicine, Shanghai 200437, China

**Keywords:** 白血病，急性, 可测量残留病灶, PCR-NGS, FLT3-ITD, Leukemia, acute, Measurable residual disease, PCR-NGS, FLT3-ITD

## Abstract

**目的:**

建立基于二代测序技术的聚合酶链反应（PCR-NGS）检测FMS样酪氨酸激酶3（FLT3）基因内部串联重复（ITD）突变的方法学，初步探索PCR-NGS检测急性白血病（AL）异基因造血干细胞移植（allo-HSCT）前FLT3-ITD的临床应用价值。

**方法:**

建立PCR-NGS检测FLT3-ITD基因突变负荷方法。PCR-NGS法检测2021年1月至2024年6月在上海交通大学医学院附属瑞金医院血液科进行allo-HSCT的65例伴有FLT3-ITD突变AL患者移植前骨髓样本，检测结果与传统定量PCR（qPCR）方法进行比较，并进行生存分析。

**结果:**

成功建立PCR-NGS检测FLT3-ITD基因突变方法，灵敏度达10^−6^，线性响应范围为10^−1^～10^−5^。63例移植前形态学缓解患者中，58例患者qPCR法检测FLT3-ITD基因突变为阴性，其中25例PCR-NGS法检测阳性［变异等位基因频率（VAF）中位值为0.629％（0.004％～26.350％）］。移植前FLT3-ITD基因突变VAF<0.1％组和≥0.1％组的2年累积复发率分别为11.2％和30.6％（*HR*＝3.159，95％*CI*：0.950～10.510，*P*＝0.048），2年无事件生存率分别为86.2％和64.5％（*HR*＝2.846，95％*CI*：0.953～8.500，*P*＝0.050）。PCR-NGS/qPCR均阴性、PCR-NGS阳性/qPCR阴性和PCR-NGS/qPCR均阳性组的2年累积复发率分别为12.5％、26.2％和28.6％（*HR*＝2.892，95％*CI*：1.122～7.451，*P*＝0.032），2年无事件生存率分别为84.4％、73.8％和57.1％（*HR*＝1.784；95％*CI*：0.880～3.615，*P*＝0.248）。移植后使用FLT3抑制剂是降低移植后复发率和延长总生存期的保护性因素。

**结论:**

成功建立PCR-NGS技术检测FLT3-ITD基因突变负荷方法。与传统qPCR检测技术相比，allo-HSCT前PCR-NGS检测FLT3-ITD突变负荷能更准确预测移植结局。

## Abstract (en)

**Objective:**

This study aimed to develop a methodology for detecting FMS-like tyrosine kinase 3 (FLT3) gene internal tandem duplication（ITD）mutation burden using polymerase chain reaction-next-generation sequencing（PCR-NGS）. The prognostic value of FLT3-ITD mutation burden detected with PCR-NGS in patients with acute leukemia（AL）before allogeneic hematopoietic cell transplantation（allo-HSCT）is investigated.

**Methods:**

This retrospective study developed a methodology for detecting FLT3-ITD mutational burden in bone marrow samples isolated from 65 patients with AL with FLT3-ITD mutations who received allo-HSCT from January 2021 to June 2024 at Ruijin Hospital. PCR-NGS was used for data analysis, and results were compared with conventional quantitative PCR（qPCR）.

**Results:**

The PCR-NGS assay demonstrated robust performance, with a sensitivity of 10^−6^. Pretransplant complete remission with FLT3-ITD negativity via qPCR was achieved in 58 patients, comprising 25 with FLT3-ITD positivity in PCR-NGS［median VAF:0.629％（0.004％–26.350％）］. The 2-year probability of relapses and event-free survival（EFS）were 11.2％ and 86.2％ for patients with FLT3-ITD VAF of <0.1％ and 30.6％ and 64.5% for those having FLT3-ITD VAF of ≥0.1％, respectively（relapse: *HR*＝3.159, 95％ *CI*: 0.950–10.510, *P*＝0.048; EFS: *HR*＝2.846, 95％ *CI*: 0.953–8.500, *P*＝0.050）. Two-year probability of relapses and EFS were 12.5％ and 84.4％ for patients with both negative PCR-NGS and qPCR, 26.2％ and 73.8％ for those with positive PCR-NGS and negative qPCR, and 28.6％ and 57.1％ for patients with both positive PCR-NGS and qPCR（relapse: *HR*＝2.892, 95％ *CI*: 1.122–7.451, *P*＝0.0321; EFS: *HR*＝1.784, 95％ *CI*: 0.880–3.615, *P*＝0.248）, respectively, before allo-HSCT. Maintenance therapy with FLT3 inhibitors is a protective factor for reduced relapse rate and better overall survival rate after allo-HSCT.

**Conclusion:**

A highly sensitive PCR-NGS assay for FLT3-ITD mutational burden has been developed. Compared with qPCR, PCR-NGS demonstrates superior sensitivity and enables more accurate prediction of posttransplant outcomes in patients with AL undergoing allo-HSCT.

FLT3基因内部串联重复（FLT3-ITD）突变是急性白血病（AL）最常见的遗传学异常之一，在急性髓系白血病（AML）中发生率为25％～30％，与不良临床特征及预后相关。FLT3-ITD突变阳性AML患者即使化疗达完全缓解（CR），复发率仍高达50％～60％[1]–[2]。异基因造血干细胞移植（allo-HSCT）是主要的巩固治疗手段[3]。

MRD检测手段主要包括多参数流式细胞术（MFC）、定量PCR（qPCR）、数字PCR（ddPCR）及基于二代测序技术的PCR（PCR-NGS）。动态监测FLT3-ITD基因突变负荷在伴有FLT3-ITD基因突变AL临床管理中具有核心意义。MFC灵敏度（10^−4^～10^−5^）较低，且易受抗原漂移干扰。基于NGS检测技术的移植前FLT3-ITD基因定量与allo-HSCT后复发率及死亡率呈显著正相关，对预后预测价值超过了FLT3-ITD等位基因比率和MFC-MRD[4]–[5]。ddPCR虽能实现绝对定量（灵敏度10^−5^），但对突变特异性引物的依赖限制了其在罕见变异中的应用[6]–[7]，且仅能检测已知突变位点，难以应对FLT3-ITD的异质性和克隆演化。PCR-NGS结合了高灵敏度（10^−6^）与广谱突变覆盖能力，可同时追踪FLT3-ITD及其他共突变，用于确定FLT3-ITD插入位点、ITD长度、等位基因比率、随时间变化的克隆优势以及每个克隆的相对丰度，尤其适用于克隆演化或突变丢失的复杂病例[8]–[9]。

尽管PCR-NGS在FLT3-ITD突变阳性AL患者MRD监测中展现潜力，仍面临检测标准化、成本效益及结果解读挑战。本研究建立了PCR-NGS检测FLT3-ITD基因突变负荷的方法学，对比qPCR检测的敏感性，探讨其临床应用价值。

## 病例与方法

一、研究对象

回顾性分析2021年1月至2024年6月在上海交通大学医学院附属瑞金医院血液科接受首次allo-HSCT 65例伴有FLT3-ITD突变的AL患者，其中AML 61例，混合表型急性白血病2例，骨髓增生异常综合征1例，早期前体T细胞急性淋巴细胞白血病1例。诊断依据WHO第5版造血和淋巴组织肿瘤分类[10]–[11]，所有患者根据骨髓细胞形态学、免疫表型分析、细胞遗传学、分子学进行诊断分型并确诊。入选标准包括：①初诊靶向测序确认伴有FLT3-ITD突变的AL；②有足够骨髓标本用于PCR-NGS检测；③移植后生存时间超过90 d；④病历资料完整。本研究经医院伦理委员会批准（批件号：院伦审【2024】08号），并在www.clinicalTrials.gov注册（NCT 06708130），所有患者均签署知情同意书。

二、预处理方案和移植物抗宿主病预防、诊断及治疗方案

预处理方案见文献[12]–[13]。急性移植物抗宿主病（aGVHD）预防以环孢素A+霉酚酸酯+小剂量甲氨蝶呤为主[14]–[15]，单倍体亲缘供者和无关全合供者加用7.5～10 mg/kg抗人胸腺细胞球蛋白。aGVHD及慢性移植物抗宿主病（cGVHD）诊断及治疗参照国际指南[16]。

三、实验方法

1. 分离骨髓单个核细胞以及提取DNA：取患者骨髓5 ml置于EDTA肝素抗凝管中，使用北京索莱宝公司生产的Ficoll分离液，提取骨髓单个核细胞。使用QIAamp DNA Blood Mini Kit（德国QIAGEN公司产品）从骨髓中提取DNA，并用Qubit™ dsDNA BR Assay Kit对DNA样品进行浓度检测。

2. 引物及探针设计：第一轮巢式PCR引物（内引物，添加NGS接头）设计如下：上游引物：5′-Illumina_Adapter + TGCTGTCCTTCCACTATACTG-3′、5′-Illumina_Adapter + CATAAGCTGTTGCGTTCATC-3′、5′-Illumina_Adapter+ACTCCTGTTTTGCTAATTCCA-3′、5′-Illumina_Adapter+CGTGCATTTTAAAGATTTTCCAATG-3′；下游引物：5′-Illumina_Adapter+GAAGGTACTAGGATCAGGTG-3′、5′-Illumina_Adapter + GCCAAATGTTTCTGCAGC-3′、5′-Illumina_Adapter + GAGTACTTCTACGTTGATTTCAGAG-3′、5′-Illumina_Adapter+CGGCTCCTCAGATAATGAG-3′。NGS接头从诺维赞公司采购AHTS Multiplex Oligos Set 4 for Illumina获得。引物由生工生物工程上海股份有限公司合成。

3. PCR体系配置及条件：第一轮反应体系（50 µl）含25 µl 2×多重PCR缓冲液、1 µl多重聚合酶、2 µl 0.4 mmol/L引物混合物、0.7～7 µg DNA模板，无核酸酶水补足体积。阳性对照为自建标准品，22 µl/反应体系；阴性即无核酸酶水。反应试剂由上海翌圣生物科技股份有限公司提供。PCR热循环参数包括初始变性步骤（95 °C，5 min）、28个扩增循环（95 °C，30 s；60 °C，90 s；72 °C，30 s）、终延伸（72 °C，5 min），12 °C保持。

使用VAHTS DNA Clean Beads（南京诺唯赞生物科技股份有限公司产品）对DNA Clean Beads（2×）1st PCR产物进行产物纯化。进行第二轮PCR引入测序接头序列及文库标签（Index），第二轮PCR体系（50 µl）含2 µl纯化PCR产物、10 µl 5×反应缓冲液、0.02 U/µl DNA聚合酶0.5 µl、0.2 mmol/L dNTP各1 µl、0.2 mmol/L P1-C 1 µl、0.2 mmol/L index-N 1 µl、34.5 µl无核酸酶水。进行PCR热循环，参数包括初始变性步骤（98 °C，1 min）、12个扩增循环（98 °C，20 s；65 °C，30 s；72 °C，20 s）、终延伸（72 °C，5 min），12 °C保持。随后进行第二轮磁珠纯化。

4. NGS文库制备及测序：使用Qubit™ dsDNA HS Assay Kit对文库进行浓度检测，目标条带一轮产物大小在100 bp，二轮产物大小在200～250 bp。使用MGIEasy环化试剂盒（中国深圳华大智造公司产品）进行环化，环化纯化完成后进行Qubit SS定量，SS定量浓度大于0.8 ng/µl。MGI测序平台进行测序，测序类型为PE150。

5. 测序数据处理分析：使用Fastp软件对这些低质量数据进行去除，将清洗后的数据与参考基因组（hg19）通过bwa软件进行比对，确定基因内插入突变位置，依据读取序列的Soft Clip、Insert size等比对信息定位ITD起点和终点。

6. 建立PCR-NGS检测FLT3-ITD基因突变方法学：空白限（LOB）建立：空白样本为6名健康正常人骨髓样本，提取gDNA分别用PCR及PCR-NGS进行检测，两种方法检测结果均为阴性，计算出PCR-NGS的空白限为0拷贝数/µl。

检测限（LOD）建立：选择3例伴有FLT3-ITD突变的AL患者骨髓样本，分别用qPCR及PCR-NGS进行检测，每个样本均重复检测3次，结果显示qPCR检测方法的检出率均为0％，而PCR-NGS检测方法变异等位基因频率（VAF）理论值为1×10^−6^时检出率为100％，VAF理论值为1×10^−7^时检出率为33.33％，故PCR-NGS检测限设为1×10^−6^。

定量限（LOQ）建立：选择2例伴有FLT3-ITD突变的AL患者骨髓样本，加入健康正常人基因组将突变频率进行稀释至1×10^−4^与1×10^−5^，应用qPCR和PCR-NGS对稀释后的不同突变频率样本进行检测，每个浓度重复6次，取每次实测的平均值，统计检测结果并计算变异系数（CV）（表1）。

**表1:** qPCR和PCR-NGS检测不同浓度FLT3-ITD基因突变

理论值（VAF）	qPCR检出率（％）	PCR-NGS检出率（％）	PCR-NGS检出均值（VAF）	变异系数（％）
1×10^−4^	0	100	1.65×10^−4^	49.0
1×10^−5^	0	100	1.67×10^−5^	84.4

**注** qPCR：定量PCR；PCR-NGS：基于二代测序技术的PCR；VAF：变异等位基因频率

线性范围：选择5例FLT3-ITD阳性，且VAF为10％左右样本，作为10^−1^样本，通过健康正常人DNA梯度稀释为10^−2^、10^−3^、10^−4^、10^−5^水平，应用PCR-NGS对稀释后的不同突变频率样本进行检测，每个浓度重复3次，取每次实测的平均值。理论稀释后的突变频率与实测突变频率的平均值呈良好的线性关系（*R*^2^＝0.9901，*P*<0.001），线性响应范围为10^−1^～10^−5^。

四、流式细胞术MRD检测方法

取患者骨髓液3 ml，全骨髓红细胞裂解法制备样本；荧光抗体标记；甲酸溶血素溶血及细胞洗涤；上机检测，根据初诊免疫表型以LAIP或DfN策略确定MRD检测方案，检测仪器：贝克曼公司Navios 10色流式细胞仪；分析软件：Kaluza。检测下限（LLOD）＝0.002％，定量下限（LLOQ）＝0.005％（检测结果<0.002％为MRD阴性）。

五、随访

通过查阅门诊、住院资料以及电话方式进行随访，截止时间为2025年11月3日。总生存（OS）期为从造血干细胞回输之日或末次随访日期；无事件生存（EFS）期为从造血干细胞回输之日至流式细胞术检测MRD为阳性、疾病复发、死亡或末次随访日期；考虑到移植相关死亡率（TRM）作为竞争风险，计算累积复发率（CIR）。

六、统计学处理

数据分析使用R 4.3.2软件进行。符合正态分布者以均数±标准差（*x*±*s*）表示，组间比较采用*t*检验。非正态分布的连续变量以中位数（范围）或四分位间距［*M*（*IQR*）］表示，使用Student's *t*检验或Mann-Whitney U检验。分类变量以频数和百分比（％）表示，采用卡方检验或Fisher确切概率法进行比较。采用Kaplan-Meier法和Log-rank检验进行生存分析。使用Cox比例风险模型进行单变量和多变量分析来确定MRD指标对预后和复发的意义。*P*<0.05为差异有统计学意义。

## 结果

一、病例特征

65例AL患者临床特征见表2，中位年龄48（16～70）岁，男32例，女33例。22例（33.8％）患者移植前使用FLT3抑制剂，中位使用时长为102（73～127）d。63例患者移植前达到CR，2例为未缓解状态。移植前39例（60.0％）患者处于CR_1_且MFC-MRD阴性。53例（81.5％）患者接受来自亲缘单倍体供者的移植物。从诊断到allo-HSCT的中位时间为137（114～168）d。61例（93.8％）患者接受清髓预处理方案。180 d Ⅱ～Ⅳ度aGVHD累积发生率为16.7％（95％ *CI*：9.8％～23.6％），2年中/重度cGVHD发生率为21.6％（95％ *CI*：14.3％～28.9％），2年CIR为17.4％（95％ *CI*：10.1％～24.7％），2年非复发死亡率为13.5％（95％ *CI*：5.3％～21.8％），2年OS率为82.9％（95％*CI*：68.9％～91.0％）。

**表2:** 65例FLT3-ITD突变阳性急性白血病患者基本临床资料

临床特征	所有患者（65例）	qPCR阴性/PCR-NGS阴性（33例）	qPCR阴性/PCR-NGS阳性（25例）	qPCR阳性/PCR-NGS阳性（7例）
年龄［岁，*M*（范围）］	48（16～70）	46（21～66）	48（20～70）	36（16～57）
性别［例（％）］				
男性	32（49.2）	16（48.5）	13（52.0）	3（42.9）
女性	33（50.8）	17（51.5）	12（48.0）	4（57.1）
移植前治疗［例（％）］				
无FLT3抑制剂	43（66.2）	22（66.7）	17（68.0）	4（57.1）
有FLT3抑制剂	22（33.8）	11（33.3）	8（32.0）	3（42.9）
移植前FLT3抑制剂中位使用时长［d，*M*（*IQR*）］	102（73～127）	108（83～129）	102（83～135）	62（59～84）
核型风险[17]［例（％）］				
良好	0（0）	0（0）	0（0）	0（0）
中等	38（58.5）	18（54.5）	14（56.0）	6（85.7）
不良	27（41.5）	15（45.5）	11（44.0）	1（14.3）
移植前疾病状态［例（％）］				
CR_1_/MFC-MRD（−）	39（60.0）	23（69.7）	14（56.0）	2（28.6）
CR_1_/MFC-MRD（+）	16（24.6）	6（18.1）	9（36.0）	1（14.3）
CR_2_/MFC-MRD（−）	4（6.2）	2（6.1）	1（4.0）	1（14.3）
CR_2_/MFC-MRD（+）	4（6.2）	2（6.1）	1（4.0）	1（14.3）
NR	2（3.0）	0（0）	0（0）	2（28.6）
移植供者来源［例（％）］				
MSD	4（6.2）	2（6.1）	2（8.0）	0（0）
MUD	8（12.3）	7（21.2）	0（0）	1（14.3）
HID	53（81.5）	24（72.7）	23（92.0）	6（85.7）
从诊断至移植时间［d，*M*（*IQR*）］	137（114～168）	138（118～174）	136（109～173）	116（92-144）
预处理强度［例（％）］				
MAC	61（93.8）	31（93.9）	23（92.0）	7（100）
RIC	4（6.2）	2（6.1）	2（8.0）	0（0）
移植后治疗［例（％）］				
无FLT3抑制剂	20（30.8）	12（36.4）	6（24.0）	2（28.6）
有FLT3抑制剂	45（69.2）	21（63.6）	19（76.0）	5（71.4）
移植后FLT3抑制剂中位使用时长［d，*M*（*IQR*）］	511（363～730）	491（424～730）	540（380～730）	321（154～551）
180 dⅡ～Ⅳ度aGVHD累积发生率［％（95％ *CI*）］	16.7（9.8～23.6）	12.1（3.5～20.7）	20.0（8.5～31.5）	28.6（4.3～52.9）
2年中/重度cGVHD累积发生率［％（95％ *CI*）］	21.6（14.3～28.9）	19.4（8.1～30.7）	23.1（10.5～35.7）	28.6（0～57.1）
2年累积复发率［％（95％ *CI*）］	17.4（10.1～24.7）	12.5（3～21.2）	26.2（9.5～38.5）	28.6（0～57.1）
2年非复发死亡率［％（95％ *CI*）］	13.5（5.3～21.8）	9.2（0～19.2）	24.0（7.3～40.7）	0（0～42.9）
2年总生存率［％（95％ *CI*）］	82.9（68.9～91.0）	88.6（81.2～100）	71.4（59.1～92.9）	100（59.0～100）

**注** qPCR：定量PCR；PCR-NGS：基于二代测序技术的PCR；CR_1_：第1次完全缓解；CR_2_：第2次完全缓解；NR：未缓解；MFC-MRD：可测量残留病灶（多参数流式细胞术法）；MSD：HLA相合同胞供者；MUD：HLA相合非亲缘供者；HID：亲缘单倍型供者；MAC：清髓预处理方案；RIC：减低强度预处理方案；aGVHD：急性移植物抗宿主病；cGVHD：慢性移植物抗宿主病

二、PCR-NGS与qPCR两种检测方法比较

63例移植前CR患者中，58例患者qPCR法检测FLT3-ITD阴性，其中25例（43.1％）PCR-NGS法检测FLT3-ITD阳性［VAF中位值为0.629％（0.004％～26.350％）］。

PCR-NGS/qPCR均阴性、PCR-NGS阳性/qPCR阴性、PCR-NGS/qPCR均阳性组的2年CIR率分别为12.5％、26.2％和28.6％，2年EFS率分别为84.4％、73.8％和57.1％。PCR-NGS/qPCR均阴性患者与PCR-NGS阳性/qPCR阴性的患者相比，2年CIR率显著降低（*HR*＝2.892，95％*CI*：1.122～7.451，*P*＝0.032）（图1）。

**图1:**
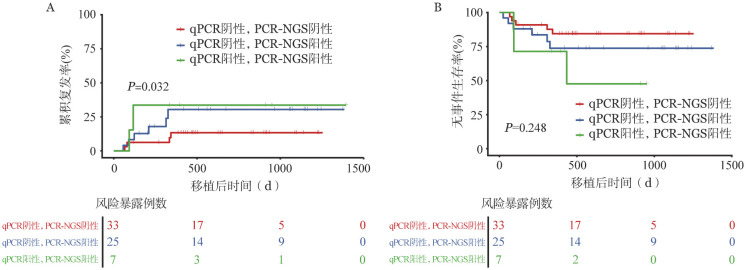
移植前不同FLT3-ITD基因突变负荷（PCR-NGS和qPCR法）急性白血病患者移植后累积复发率（A）和无事件生存率（B）比较 **注** qPCR：定量PCR；PCR-NGS：基于二代测序技术的PCR

三、PCR-NGS检测FLT3-ITD基因VAF水平及ITD长度对生存的影响

将移植前FLT3-ITD阳性（PCR-NGS法）患者分为低水平（VAF<0.1％）和高水平FLT3-ITD组（VAF≥0.1％）。两组2年CIR率分别为11.2％和30.6％（*HR*＝3.159，95％*CI*：0.950～10.510，*P*＝0.048），2年EFS率分别为86.2％和64.5％（*HR*＝2.846，95％*CI*：0.953～8.500，*P*＝0.050）（图2）。

**图2:**
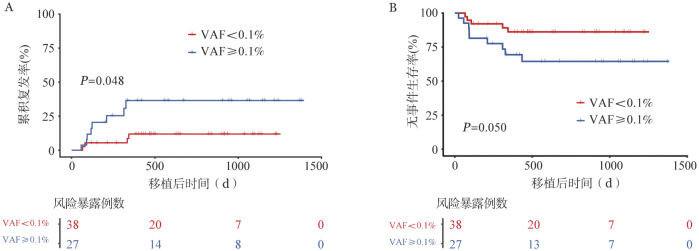
基于二代测序技术的PCR检测移植前不同FLT3-ITD基因突变负荷急性白血病患者的累积复发率（A）和无事件生存率（B）比较 **注** VAF：变异等位基因频率

根据FLT3-ITD不同片段长度，将患者分为短FLT3-ITD片段组（ITD长度<48 bp，11例）和长FLT3-ITD片段组（ITD长度≥ 48 bp，21例）。两组2年CIR率分别为19.2％和29.2％（*HR*＝1.581，95％*CI*：0.318～7.871，*P*＝0.572），2年EFS率分别为80.8％和64.9％（*HR*＝1.679，95％*CI*：0.348～8.104，*P*＝0.514）（图3）。

**图3:**
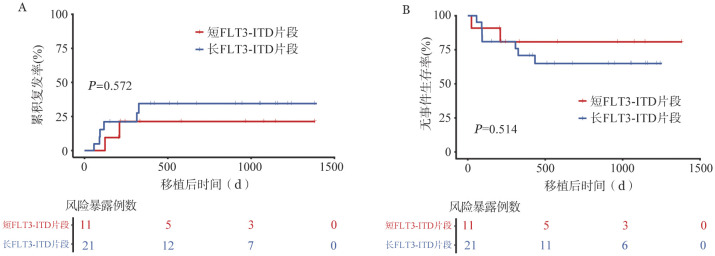
基于二代测序技术的PCR检测不同FLT3-ITD基因片段长度急性白血病患者的累积复发率（A）和无事件生存率（B）比较

四、FLT3-ITD片段克隆演变

移植前50.8％（33/65）的患者FLT3-ITD为阴性（PCR-NGS法）。1例患者移植前原有ITD片段消失，但出现新ITD片段。13.8％（9/65）的患者在初诊至移植前出现ITD插入位点或ITD长度发生改变。2例（3.1％）在原有ITD的基础上出现新的ITD片段。30.8％（20/65）的患者在初诊及移植前检测到相同ITD片段。18.5％（12/65）的患者在初诊至移植前出现ITD克隆演变，其中16.7％（2/12）的患者移植前使用过FLT3抑制剂。

PCR-NGS法对6例移植后MFC-MRD由阴性转为阳性患者进行检测，其中3例仍为FLT3-ITD阳性，3例FLT3-ITD阴性。1例初诊时FLT3-ITD阳性，移植前及移植后MFC-MRD转阳时均未检测到FLT3-ITD片段；2例初诊及移植前检测到FLT3-ITD，移植后MFC-MRD转阳时未检测到相应的ITD片段；2例在初诊、移植前及移植后MFC-MRD转阳时均检测到相同ITD片段，2例患者移植前均使用过FLT3抑制剂；1例在移植前及移植后MFC-MRD转阳时均出现新的ITD片段。

五、预后因素分析

对CIR、EFS、OS进行单因素分析，纳入因素包括年龄、移植前后有无使用FLT3抑制剂、移植后FLT3抑制剂使用时长、核型风险、移植供者来源、核型风险、是否合并Ⅱ～Ⅳ度aGVHD、有无合并中/重度cGVHD、移植前PCR-NGS评估MRD、FLT3-ITD基因VAF值水平、FLT3-ITD片段长度。结果显示，移植后使用FLT3抑制剂是降低移植后复发率（*HR*＝0.06，95％*CI*：0.01～0.30，*P*<0.001）和延长生存期的保护性因素（EFS：*HR*＝0.12，95％*CI*：0.04～0.40，*P*<0.001；OS：*HR*＝0.04，95％*CI*：0.01～0.34，*P*＝0.003）。

## 讨论

近年来，FLT3抑制剂的涌现为伴有FLT3基因突变AL临床治疗带来了革命性改变。吉瑞替尼（Gilteritinib）[18]–[19]、米哚妥林（Midostaurin）[20]和奎扎替尼（Quizartinib）[21]–[22]等代表性药物已获批用于临床，显著改善了生存结局。ADMIRAL研究中吉瑞替尼治疗复发/难治性（R/R）AML患者OS较传统化疗延长近一倍（9.3个月对5.6个月）[23]。吉瑞替尼使R/R AML患者达到深度缓解，有更多机会桥接移植[23]–[24]。奎扎替尼也被用于R/R AML的挽救治疗[25]。Erba等[26]发现奎扎替尼联合标准化疗的3年CIR为30％，中位OS期31.9个月，显著优于安慰剂组。allo-HSCT仍然是伴有FLT3-ITD突变AML患者最佳巩固治疗方式，相比于化疗组可提供20％～30％额外生存获益，移植后2年无复发生存（RFS）率达到50％～55％。在QuANTUM-First研究中，如果在allo-HSCT前进行删失，奎扎替尼仅能提供微弱的生存优势（20.8个月对12.9个月，*HR*＝0.75）。但将移植纳入治疗分析后，奎扎替尼组的OS期（31.9个月对15.1个月，*P*＝0.032）和CIR（30％对42％）显著优于安慰剂组。同样，在米哚妥林的系列研究中也有类似的临床结果[27]。

FLT3-ITD MRD动态监测对移植后预后评估至关重要。基于NGS技术的MRD检测可识别复发高风险患者、指导早期干预（如调整免疫抑制剂或加用FLT3抑制剂）以延缓复发，例如吉瑞替尼在抢先治疗中的应用已显示临床获益[28]。MORPHO研究证实，吉瑞替尼维持治疗仅能改善allo-HSCT后围移植期FLT3-ITD MRD阳性患者结局，但MRD阴性患者无法获益，反而增加药物毒性及经济负担等。此项研究表明，对FLT3-ITD阳性AML患者进行移植前后FLT3定量检测不仅有助于预后评估，还能指导FLT3抑制剂的移植后分层应用[19]。我们的数据证实，采用更高灵敏度PCR-NGS技术对allo-HSCT前FLT3-ITD基因突变负荷进行定量分析有重要预后价值。PCR-NGS和qPCR检测均阴性患者相比于qPCR阴性但PCR-NGS阳性患者有更高的移植后长期生存率和更低的复发率（*P*<0.05）。另外FLT3-ITD基因突变负荷阈值对MRD监测也有影响，动态变化的FLT3-ITD突变丰度增加了MRD监测难度。FLT3-ITD检测VAF<0.1％（低于qPCR检测阈值）的患者，移植后复发率为11.2％，提示即使移植前检测到极低水平FLT3-ITD基因突变负荷仍与不良临床转归显著相关。

FLT3-ITD突变会导致FLT3蛋白的近膜结构域或酪氨酸激酶1结构域发生不同程度的延长[29]。FLT3-ITD片段长度所产生的影响存在争议，但可能与更多的自身磷酸化有关，进而导致生存结局不良。近膜结构域中存在长FLT3-ITD片段AML患者OS期较短，可能与更高程度的组成型激酶激活相关，从而导致更具侵袭性的表型[29]–[30]。一项荟萃分析结果显示，与短FLT3-ITD长度相比，长FLT3-ITD长度具有更高死亡风险[31]。我们的研究也证实，移植前携带长FLT3-ITD片段患者移植后复发率更高，EFS期更短。

FLT3-ITD具有异质性和克隆演化的特征，其突变序列和长度存在显著的异质性，且患者体内可能存在多个携带不同FLT3-ITD突变的亚克隆，增加了对FLT3-ITD突变准确检测和动态监测的挑战。一项米哚妥林治疗后FLT3-ITD阳性AML患者克隆演变模式研究显示，接受米哚妥林治疗患者中，22％（12/54）在疾病进展时出现部分克隆丢失、获得新FLT3-ITD变异或FLT3-ITD位置和插入长度发生了转换等克隆演变[32]。本研究中12例患者（18.5％）在初诊至移植前观察到了FLT3-ITD片段变化，其中2例患者移植前使用过FLT3抑制剂。使用FLT3抑制剂移植前FLT3-ITD转阴比例与未使用过FLT3抑制剂比例相近（50％比51％）。

FLT3-ITD阳性AML患者获益于移植后FLT3抑制剂维持治疗已逐渐获得认可。美国国家综合癌症网络（NCCN）指南[33]及欧洲血液及骨髓移植学会（EBMT）[34]均推荐移植后使用FLT3抑制剂进行维持治疗。南方医院刘启发教授团队开展的开放标签随机对照Ⅲ期研究[35]以及事后分析[36]结果均显示索拉非尼能显著降低移植后1年CIR（*HR*＝0.25，*P*＝0.001）。SORMAIN研究中，83例FLT3-ITD阳性患者allo-HSCT后索拉非尼维持治疗2年，2年RFS率显著高于安慰剂组（85％对53.3％，*P*＝0.01）[37]。MORPHO研究结果显示移植后吉瑞替尼维持治疗仅能降低围移植期FLT3-ITD MRD阳性患者的复发率[38]。我们的研究结果显示，移植后使用FLT3抑制剂是降低移植后复发率和延长OS期的保护性因素（*P*<0.05）。EBMT指南推荐移植后FLT3抑制剂最佳维持时间为2年[39]。但也有研究发现，FLT3-ITD阳性AML移植后复发在移植后3年内达到平台期，推荐FLT3抑制剂应维持满3年[40]。

综上，基于PCR-NGS平台FLT3-ITD动态监测体系不仅完善了现有预后评估系统，更为重要的是为建立基于分子标志物的精准治疗策略提供了理论依据，这对改善移植患者长期生存具有重要转化医学意义。本研究样本量较小，随访期内发生的事件数少，不能进行有效的多因素分析。未来研究首先需要扩大样本量，进行多中心前瞻研究设计，着重探讨通过该技术筛选的FLT3-ITD MRD阳性患者群体，能否通过FLT3抑制剂等靶向治疗获得更优生存获益，特别是在移植前后联合治疗模式中的应用价值。
